# Virgibacillus tibetensis sp. nov., isolated from salt lake on the Tibetan plateau of China

**DOI:** 10.1099/ijsem.0.006525

**Published:** 2024-09-23

**Authors:** Dongyang Li, Dorji Phurbu, Xuan Zhang, Zi-Xuan Liu, Rui Wang, Yan-Yan Zheng, Yu-Guang Zhou, Ya-Jing Yu, Lu Xue, Ai-Hua Li

**Affiliations:** 1 China General Microbiological Culture Collection Center (CGMCC), Institute of Microbiology, Chinese Academy of Sciences, Beijing 100101, PR China; 2 School of Biotechnology and Food Science, Tianjin University of Commerce, Tianjin, PR China; 3 Tibet Plateau Key Laboratory of Mycology, Tibet Plateau Institute of Biology, Lhasa, Tibet 850001, PR China; 4 Tianjin Institute of Industrial Biotechnology, Chinese Academy of Sciences, Tianjin 300308, PR China

**Keywords:** *Bacillaceae*, salt lake, Tibetan Plateau, *Virgibacillus*

## Abstract

One bacterial strain, designated as C22-A2^T^, was isolated from Lake LungmuCo in Tibet. Cells of strain C22-A2^T^ were long rod-shaped, Gram-stain-negative, non-spore-forming, with positive catalase and oxidase activity. Optimal growth occurred at 20–25 °C, pH 8.0 and with 3.0–7.0% (w/v) NaCl. Phylogenetic analysis of 16S rRNA gene and whole genome sequences revealed that strain C22-A2^T^ belonged to the genus *Virgibacillus*, showing the highest 16S rRNA gene similarity to *Virgibacillus halodenitrificans* DSM 10037^T^ (97.6%). The average nucleotide identity values between strain C22-A2^T^ and the type strains of related species in the genus *Virgibacillus* were less than 74.4% and the digital DNA–DNA hybridization values were less than 20.2%, both below the species delineation thresholds of 95 and 70% respectively. The genome analysis revealed that strain C22-A2^T^ harboured genes responsible for osmotic and oxidative stress, enabling it to adapt to its surrounding environment. In terms of biochemical and physiological characteristics, strain C22-A2^T^ shared similar characteristics with the genus *Virgibacillus,* including the predominant cellular fatty acid anteiso-C_15 : 0_, the major respiratory quinone MK-7, as well as the polar lipids phosphatidylglycerol and diphosphatidylglycerol. Based on the comprehensive analysis of phylogenetic, phylogenomic, morphological, physiological and biochemical characteristics, strain C22-A2^T^ is proposed to represent a novel species of the genus *Virgibacillus*, named as *Virgibacillus tibetensis* sp. nov. (=CGMCC 1.19202^T^=KCTC 43426^T^).

## Introduction

The genus *Virgibacillus*, belonging to the family *Bacillaceae*, was first described by Heyndrickx *et al* in 1998 [1]. Up to the time of writing, there were 34 validly published species in the genus according to LSPN database. The genus *Virgibacillus* is distributed worldwide, including in China, Korea, Algeria and Canada [2,4]. The majority of species in the genus *Virgibacillus* are found in high-salinity environments, such as salt lakes and salt-fermented seafood [5]. *Virgibacillus kekensis* was isolated from a salt lake in China [3], *Virgibacillus chiguensis* was isolated from commercial saltern located in southern Taiwan and *Virgibacillus dokdonensis* was isolated from the edge of the East Sea in Korea [4,6]. *Virgibacillus halodenitrificans*, isolated from a solar saltern, was first proposed as *Bacillus halodenitrificans* by Denariaz *et al.* [7], and verified as *Virgibacillus halodenitrificans* based on the phylogenetic analysis by Yoon *et al.* [8].

In this research, water samples were collected from LungmuCo lake to investigate the bacterial resources of salt lakes in Tibet. Strain C22-A2^T^ was isolated and described as a novel species of the genus *Virgibacillus* based on phylogenetic, genomic and polyphasic taxonomic results.

## Isolation and growth conditions

LungmuCo lake is a high-altitude salt lake located in Tibet, PR China. Strains were isolated from the water sample by 10-fold dilution plating and incubated on MA (marine agar 2216) plates at 25 °C. After 14 days cultivation, single colonies appeared and purified by lining separation on MA plates. Strain C22-A2^T^ was obtained for further research.

To optimize the growth conditions of strain C22-A2^T^, a series of experiments were carried out. Growth of strain C22-A2^T^ on Luria–Bertani (LB) agar, tryptic soy agar (TSA), peptone–yeast–glucose (PYG) agar, marine agar 2216 (MA), Reasoner’s 2A (R2A) agar and nutrient agar (NA) was assessed. Growth with different NaCl concentration (with 1% intervals; w/v) in PYG broth was assessed, cultivating at 25 °C for 7 days. Growth at different pH values ranging from pH 5 to 12 with intervals of 1 pH unit were tested. The pH value was adjusted by 1 M acetate–sodium aetate buffer (for pH 5.0), 200 mM NaHCO_3_–Na_2_CO_3_ buffer (for pH 6.0–8.0), 200 mM Na_2_HPO_4_–NaH_2_PO_4_ buffer (for pH 9.0–10.0), Na_2_HPO_4_–NaOH (for pH 11.0) and KCl/NaOH (for pH 12.0), respectively. Growth at different temperatures (4, 10, 20, 25, 30, 37, 45, 50 °C) was measured via cultivation in PYG broth with 5% NaCl (w/v) for 7 days. Anaerobic growth was tested on PYG plates with 5% NaCl (w/v) under an anaerobic environment created by using Anaeropack. Results were read after 3 and 7 days, respectively.

The results showed that strain C22-A2^T^ could grow on LB, R2A, MA, PYG and NA media. Growth occurred at 4–30 °C, at pH 7.0–10.0 and with 1–17% (w/v) NaCl. The optimum growth conditions for strain C22-A2^T^ were at 20–25 °C, pH 8.0 and with 3–7% (w/v) NaCl. The strain C22-A2^T^ could not grow under anaerobic condictions.

## Phylogenetic analyses based on 16S rRNA

For the phylogenetic analysis of strain C22-A2^T^, genomic DNA was extracted using the Genomic DNA Rapid Isolation Kit for bacterial cells (BioDev-Tech) and the 16S rRNA gene sequence was amplified using the universal primers 27F and 1492R [9,10]. A nearly complete 16S rRNA gene sequence (1426 bp) was acquired and aligned using the GenBank and EzBio-Cloud databases [11]. Sequences of the related taxa were acquired from the EzBioCloud database and the phylogenetic analysis based on 16S rRNA gene sequences was conducted by mega X [12]. Multiple alignments were performed with Clustal W [13]. Evolutionary distance matrices were calculated using the algorithm of Kimura’s two-parameter model [14]. Phylogenetic trees were reconstructed using the neighbour-joining [15], maximum-likelihood [16] and maximum-parsimony methods [17] respectively, with the topology of the phylogenetic tree evaluated based on 1000 replications.

**Fig. 1.**
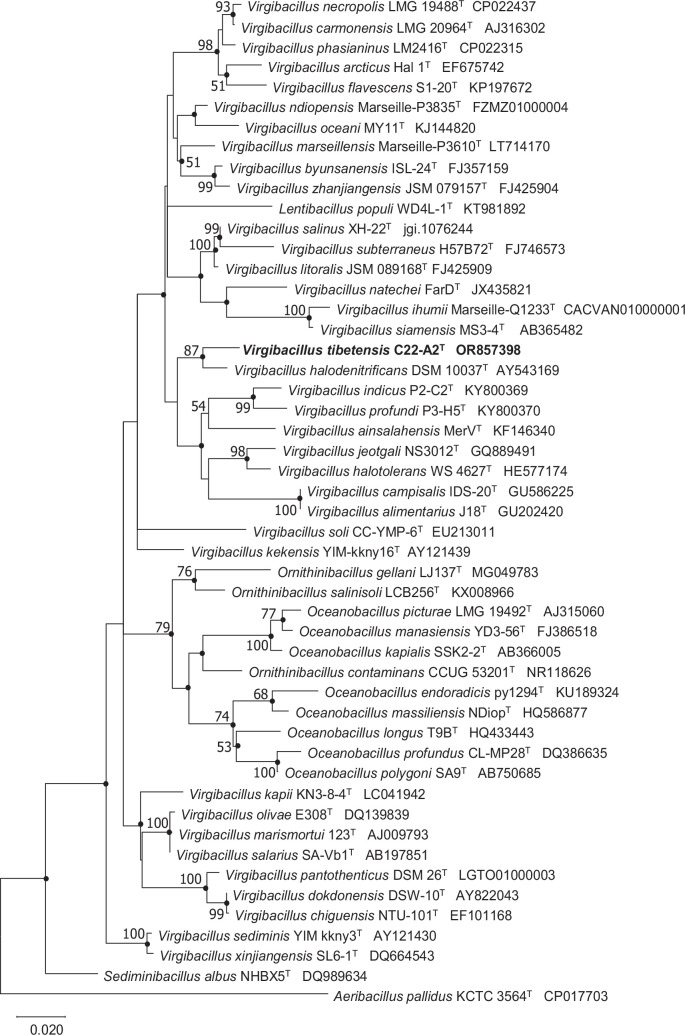
Maximum-likelihood phylogenetic tree based on 16S rRNA gene sequences of strain C22-A2^T^ and related taxa. Bootstrap values were expressed as percentages of 1000 replications and shown at branching nodes (>50%). GenBank accession numbers were shown in parentheses. The filled circles indicate nodes recovered using the neighbour-joining method and maximum-parsimony methods. Bar, 0.02 substitution per nucleotide position. *Aeribacillus pallidus* KCTC 3564^T^ was used as an outgroup.

On the basis of 16S rRNA gene sequence comparisons using the GenBank and EzBioCloud databases, strain C22-A2^T^ showed the closest relationships with *Virgibacillus halodenitrificans* DSM 10037^T^, Virgibacillus jeotgali*Virgibacillus jeotgali* NS3012^T^, and *Virgibacillus salinus* XH-22^T^, with similarity values of 97.6, 97.4 and 97.2% respectively. The maximum-likelihood tree, reconstructed with the K2 +G+I model, demonstrated that strain C22-A2^T^ forms a distinct branch with *V. halodenitrificans* DSM 10037^T^, and then clusters with other species of the genus *Virgibacillus* (Fig. 1). The neighbour-joining tree showed similar phylogenetic relationships to those shown in the maximum-parsimony tree. These results indicated that strain C22-A2^T^ represented a novel species of genus *Virgibacillus*.

## Genome analysis

The genome of strain C22-A2^T^ was sequenced via the Illumina HiSeq X platform at the Majorbio Co., Ltd (Shanghai, PR China). The raw data was acquired by signal conversion and then statistical analysis was carried out to count and control base sequences. The clean data were uploaded to the BV-BRC platform version 3.32.13a (https://www.bv-brc.org/), and assembled from scratch using Unicycler version 0.4.8 in the Genome Assembly tool [18]. Phylogenomic analysis of strain C22-A2^T^ together with closely related species was further performed by Bacterial genome tree tool (BV-BRC platform). The phylogenomic tree was reconstructed based on single-copy genes and the number of genes was set as 1000 [19]. The average nucleotide identity (ANI) values were calculated using the ANI Calculator tool (www.ezbiocloud.net/tool/ani) [20]. Digital DNA–DNA hybridization (dDDH) values were calculated via the Genome-to-Genome Distance Calculator 3.0 (https://ggdc.dsmz.de/ggdc.php) with formula 2 [21]. The average amino acid identity (AAI) values were acquired by estimating the genomic datasets of proteins via AAI Calculator tool (http://enve-omics.ce.gatech.edu/aai/index) [22].

**Fig. 2.**
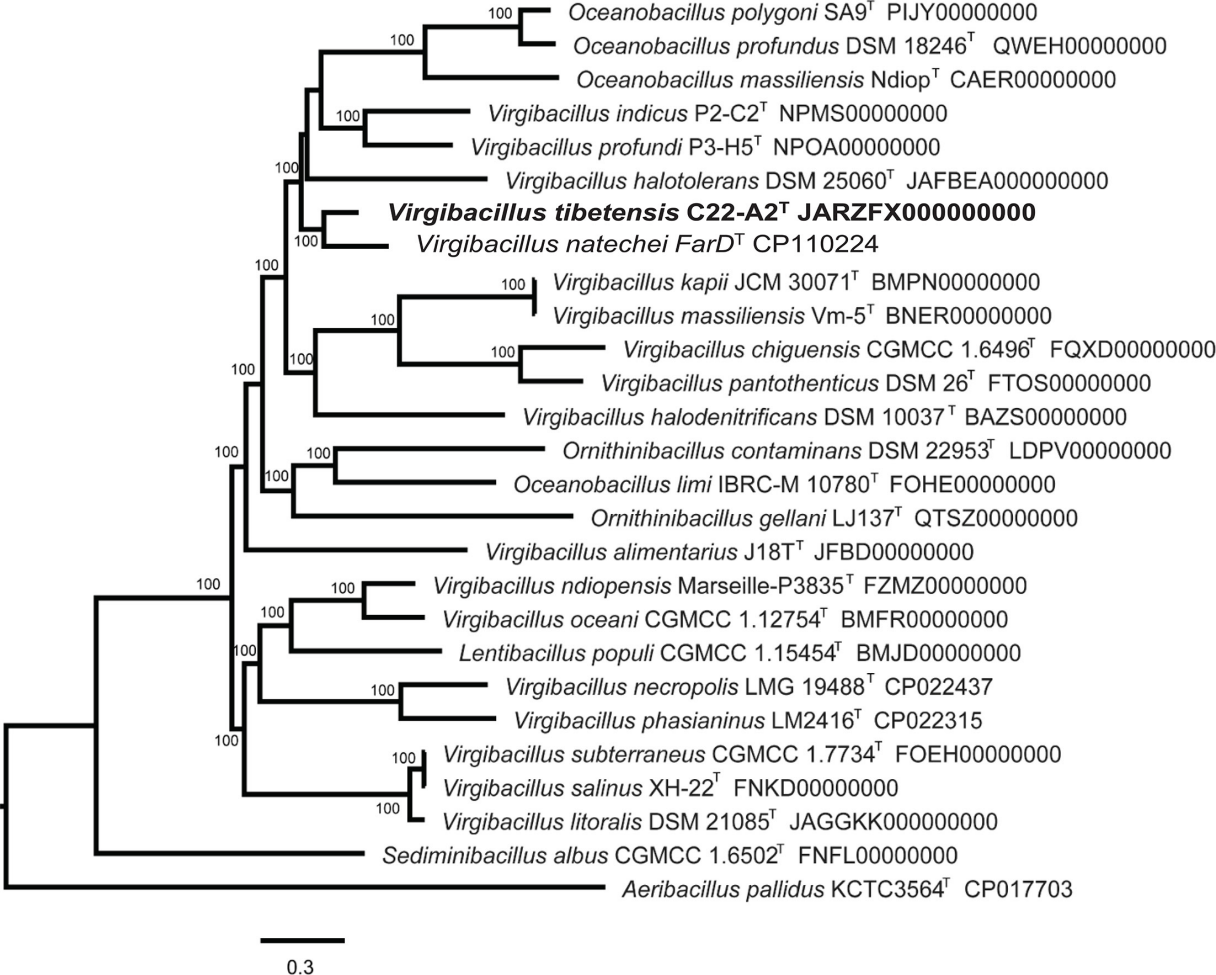
The phylogenomic tree of strain C22-A2^T^ and type strains of related species of genus *Virgibacillus* reconstructed by Bacterial genome tree tool (BV-BRC platform), based on single-copy genes and the number of genes was set as 1000. Bar, 0.3 nucleotide substitution rate (Knuc) units.

The genome of strain C22-A2^T^ was 4.29 Mb, consisted of 43 contigs, with an average short read coverage of 269-fold and an N50 value of 318.2 kb. The genomic DNA G+C content was 37.5 mol%, which was calculated based genomic bioinformatics. The draft genome of strain C22-A2^T^ was submitted to DDBJ/ENA/GenBank under the accession number JARZFX000000000. The phylogenomic tree was reconstructed based on 446 single-copy genes. As shown in Fig. 2, strain C22-A2^T^ clustered with *Virgibacillus natechei* FarD^T^ as a clade. This branch further gathered other species of the genus *Virgibacillus* and *Oceanobacillus*. Based on the above results, we selected five type strains of related species of the genus *Virgibacillus* as reference strains, including *Virgibacillus halodenitrificans* DSM 10037^T^, *Virgibacillus salinus* XH-22^T^ [23], *Virgibacillus indicus* P2-C2^T^ [24], *Virgibacillus profoundi* P3-H5^T^ [24] and *Virgibacillus natechei* FarD^T^ [25]. The dDDH values (computed with GGDC 3.0 and formula 2) between novel isolate C22-A2^T^ and the five reference strains were 19.2–20.2%, apparently lower than the threshold of 70%. ANI values of strain C22-A2^T^ with the five related species ranged from 72.3 to 74.4%, which were also below the species-delimiting threshold of 95–96%. In addition, AAI values between strain C22-A2^T^ and the reference strains were calculated as 69.5–72.5%, which were also below the proposed species-delimiting criteria of 95–96% (Table 1). These results further confirmed that strain C22-A2^T^ represents a novel species of the genus *Virgibacillus*.

**Table 1.** Comparison based on genome information between strain C22-A2^T^ and the type strains of related species of genus *Virgibacillus*

Reference strain	Accessions	16S rRNA gene identity (%)	ANI (%)	DDH (%)	AAI (%)
*V. halodenitrificans* DSM 10037^T^	BAZS00000000	97.6	72.3	19.2	69.9
*V. salinus* XH-22^T^	FNKD00000000	97.2	72.3	20.2	69.5
*V. indicus* P2-C2^T^	NPMS00000000	96.2	73.4	19.2	72.3
*V. profoundi* P3-H5^T^	NPOA00000000	96.2	74.4	19.9	72.5
*V. natechei* FarD^T^	CP110224	95.9	73.4	19.8	69.7

## Annotation of gene function

The draft genome was acquired and annotated via the Genome Annotation tool (BV-BRC platform). Functional annotation of the genome was performed by Rapid Annotation using Subsystems Technology (rast) and visualized with the seed viewer [26,27]. Biosynthesis gene clusters were predicted using Antibiotic and Secondary Metabolite Analysis Shell (antiSMASH) in order to analyse the bioactive potential of strain C22-A2^T^ [28].

Strain C22-A2^T^ was predicted to harbour 4197 protein-coding genes (CDS), 61 tRNA and four rRNA genes, respectively. The 16S rRNA gene sequence acquired from the genome was compared with the nearly full-length 16S rRNA gene sequenced by the Sanger method, and the result showed 99.9% similarity (1425/1426 nt). The protein analysis showed that there were 83 hypothetical proteins. Genes related to antibiotic resistance and virulence factors in the genome of strain C22-A2^T^ were identified using various databases. Genes including *alr*, *ddl*, *EF-G*, *EF-Tu*, *folA*, *dfr*, *folP*, *gyrA*, *gyrB*, *inhA*, *fabI*, *kasA*, *rpoB* and *rpoC* were assigned into antibiotic targets in susceptible species [29]. More details were presented in Table S1, available in the online version of this article.

According to the seed viewer, 1432 genes were annotated into the subsystem category in strain C22-A2^T^ (Fig. S1). The major subsystem features of strain C22-A2^T^ were classified as amino acids and derivatives (315), followed by protein metabolism (189), carbohydrates (158), and cofactors, vitamins, prosthetic groups and pigments (126). In addition, fatty acids, lipids, and isoprenoids (82), DNA metabolism (67), membrane transport (61), and stress response (52) also represented large numbers of genes.

For the secondary metabolite analysis, the genome of strain C22-A2^T^ was uploaded to the antiSMASH website. Lasso peptides are a structurally interesting and pharmacologically relevant class of RiPPs with antimicrobial, antiviral, receptor antagonistic, or enzyme inhibitory activities [30,31]. A lasso peptide synthesis gene cluster was identified in strain C22-A2^T^, showing 80% similarity to that of the paeninodin synthesis gene cluster acquired from *Paenibacillus dendritiformis* C454 [32]. The gene cluster for ectoine synthesis was also discovered in the C22-A2^T^ genome, indicating its ability to adapt to a high saline environment. Micro-organisms accumulate or release specific organic osmolytes, such as the amino acid proline, the trimethylammonium compound glycine betaine and the tetrahydropyrimidine (ectoine), to survive hyperosmotic conditions [33]. Considering the saline lake environment where strain C22-A2^T^ inhabited, it was believed that strain C22-A2^T^ also adjusted osmotic pressure through the synthesis of ectione.

## Morphological, biochemical and physiological tests

Cell morphology was observed by using light microscope and transmission electron microscope (JEM1400, jeol) after 48h cultivation on MA at 25 °C. The motility of strain C22-A2^T^ was assessed by inoculating it into semi-solid MA agar medium (0.5% agar), resulting in the observation of a cloudy growth trace. Tests of Gram-staining and the presence of DNase, catalase and oxidase activities were performed according to the standard methods [34]. The physiological and carbon source utilization tests were performed using the GEN III MicroPlate system (Biolog) and API ZYM, 20E, 20NE and 50CH (bioMérieux) test strips according to the manufacturers’ instructions. Hydrolysis of substrates (5.0% casein, 1.0% cellulose, 1.0% Tween 20, 1.0% Tween 80, 1.0% starch, w/v) were evaluated on MA plates at 25 °C for 7 days. Reference strains, including *V. halodenitrificans* DSM 10037^T^, *V. salinus* XH-22^T^ [23], *V. indicus* P2-C2^T^ [24], *V. profoundi* P3-H5^T^ [24] and *V. natechei* FarD^T^ [25] were selected for comparison.

**Fig. 3.**
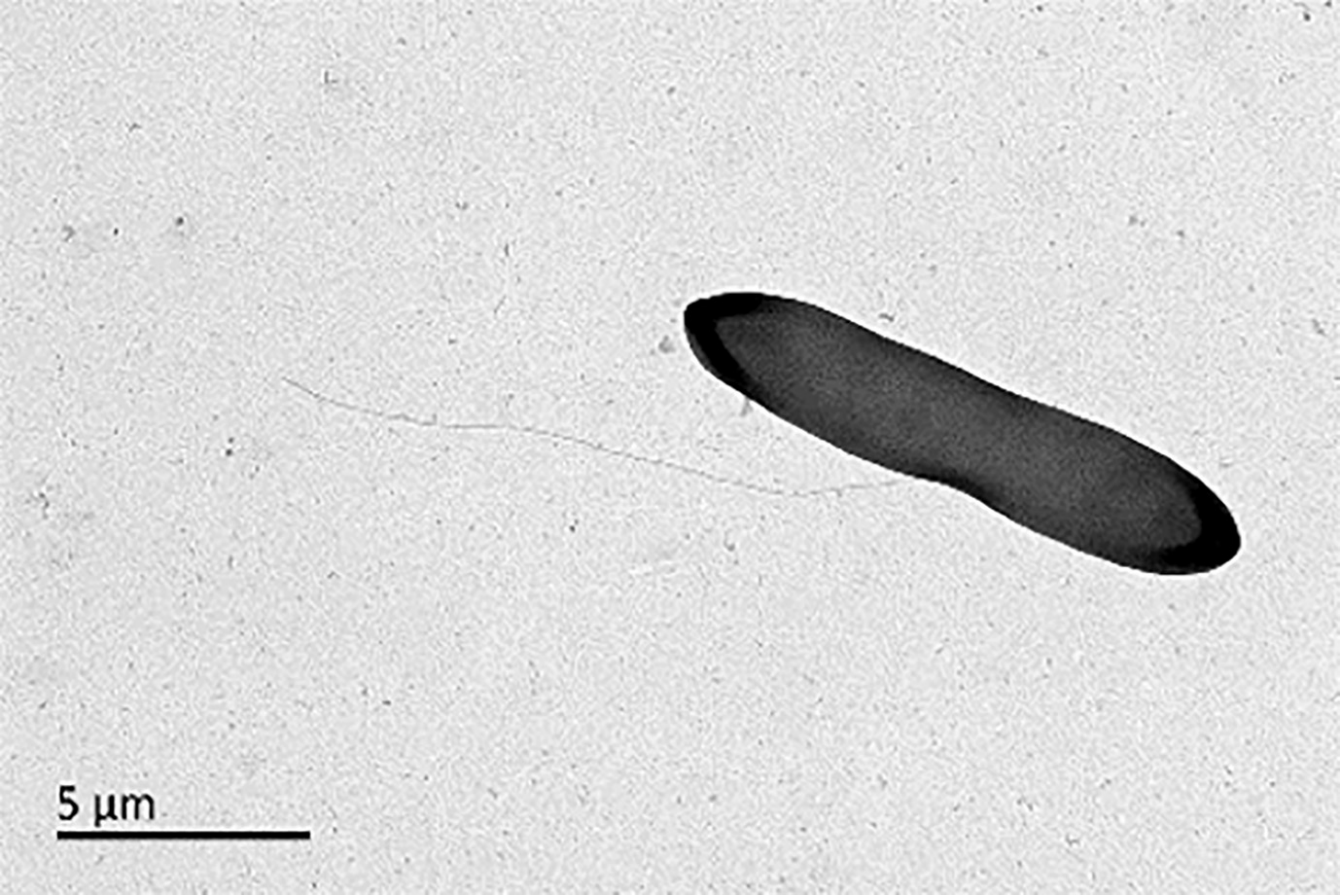
Transmission electron microscope image of strain C22-A2^T^ cultivated on MA at 25 °C for 48 h.

The colonies were non-transparent white-coloured. Cells of strain C22-A2^T^ were long rod-shaped and flagella were observed (Fig. 3). Each cell was about 6.0–12 µm long and 2.0–3.0 µm wide. Strain C22-A2^T^ was Gram-stain-negative and DNase-negative. Catalase and oxidase activity were positive. Starch could be hydrolysed, while the rest of tested substrates (casein, cellulose, Tween 20, Tween 80) could not. Based on the comparison of physiological characteristics, there were significant differences between strain C22-A2^T^ and the reference strains. Firstly, strain C22-A2^T^ showed moderate carbon source utilization capacity, unlike *V. halodenitrificans* DSM 10037^T^, which could use a variety of carbon sources, and *V. salinus* XH-22^T^, which could only use a few carbon sources. The ability to utilize *N*-acetyl-d-galactosamine, a-d-glucose, d-mannose, d-frucose, d-mannitol and l-serine as sole carbon resources, distinguished them from each other clearly. Secondly, C22-A2^T^ could hydrolyse gelatin, PNPG and ONPG, exhibiting the same characteristics as *V. halodenitrificans* DSM 10037^T^, while differentiating it from other species. Thirdly, it is worth noting that strain C22-A2^T^ could not produce acids from any carbon sources from the API 50CH kit, while *V. halodenitrificans* DSM 10037^T^ could produce acid from glycerol, d-fructose, d-mannose and so on. Other morphological, biochemical and physiological properties are summarized in Table 2 and in the description of the new species.

**Table 2.** Differential characteristics between the novel strain and the most related phylogenetic neighbours of genus *Virgibacillus* Strain: 1, C22-A2^T^; 2, *V. halodenitrificans* DSM 10037^T^; 3, *V. salinus* XH-22^T^; 4, *V. indicus* P2-C2^T^; 5, *V. profoundi* P3-H5^T^. +, Positive; −, negative; w, weakly positive; nd, not determined. All of the data were acquired from this experiment except where indicated otherwise.

Characteristic	1	2	3	4	5
pH range (optimum) for growth	7.0–10.0 (8.0)	6.0–9.5 (7.0–7.5)	6.0–10.0 (7.5)	6.0–10.0 (7.5–8.0)	6.5–9.0 (7.0)
Temperature range (optimum) for growth (°C)	4–30 (20–25)	4.0–42 (35)	4.0–40 (35–37)	4–40 (20–25)	4–30 (15–25)
NaCl range (optimum) for growth (%, w/v)	1.0–17 (3.0–7.0)	0.5–20 (2.0–6.0)	1.0–16 (2.0–4.0)	0–16 (2.0–4.0)	1.0–14 (4.0–6.0)
Utilization of substrates (Biolog GENIII):					
*N*-Acetyl-d-galactosamine	+	w	−	−	−
α-d-Glucose	−	+	−	+	w
d-Mannose	−	+	−	+	+
d-Frucose	−	+	w	w	+
d-Mannitol	+	+	−	−	+
l-Serine	+	+	−	+	+
Enzyme activity (API ZYM):					
Leucine arylamidase	+	+	w	+	−
*N*-Acetyl-*β*-glucosaminidase	−	+	−	−	−
Reactions (API 20E and 20NE):					
Urease	−	−	−	−	+
Hydrolysis of gelatin	+	+	−	+	+
Para-nitrophenyl-*β*-d-galactopyranosidase, PNPG	+	+	−	−	+
Orthon nitrophenyl-*β*-d-galactopyranosidase, ONPG	+	+	−	−	+
Tryptophane deaminase	−	−	−	−	−

For fatty acid profile analysis, strain C22-A2^T^ and the reference strains were cultured on MA at 25 °C for 48 h. The cells were harvested, saponified, methylated and extracted according to the standard protocol [35]. The extracted cellular fatty acids were further identified and quantified by the Sherlock Microbial Identification System (midi) using a gas chromatograph (Agilent 6890 N) [36]. For polar lipid and respiratory quinone tests, strain C22-A2^T^ was cultured in MA broth with 200 r.p.m. at 25 °C for 48 h. Polar lipids were extracted and separated according to the previous method and identified by two-dimensional silica-gel TLC [37]. The mixture of chloroform–methanol–water (65 : 25 : 4, v/v/v) was used for the first direction and a mixture of chloroform–acetic acid–methanol–water (80 : 15 : 12 : 4, v/v/v) for the second direction. Molybdatophosphoric acid, molybdenum blue, ninhydrin and anisaldehyde reagent were used to detect the total polar lipid, phospholipids, aminolipids and glycolipids, respectively. The respiratory quinones was extracted, separated and purified following the previous description [38]. The specific components were analysed by HPLC [39].

The primary cellular fatty acid of strain C22-A2^T^ was anteiso-C_15 : 0_ (64.2%), which was in line with the fatty acid profiles of members if the genus *Virgibacillus*. While the contents of summed feature 4 (iso-C_17 : 1_ I/anteiso-C_17 : 1_), anteiso-C_17 : 0_, iso-C_14 : 0_, iso-C_15 : 0_, iso-C_16 : 0_ and C_16 : 0_ were significantly different among members of the genus *Virgibacillus*. More details are presented in Table 3. In terms of the respiratory quinone, MK-7 was obviously the predominant component, which was in accordance with the characteristics of the genus *Virgibacillus* [8]. The major polar lipids were phosphatidylglycerol and diphosphatidylglycerol, which also matched the characteristics of the genus *Virgibacillus*. In addition, two unidentified lipids could distinguish strain C22-A2^T^ from other species of the genus *Virgibacillus* (Fig. S2).

**Table 3.** Whole-cell fatty acid profiles of the novel isolate C22-A2^T^ and type strains of related species of genus *Virgibacillus* Strains: 1, C22-A2^T^; 2, *V. halodenitrificans* DSM 10037^T^; 3, *V. salinus* XH-22^T^; 4, *V. indicus* P2-C2^T^; 5, *V. profoundi* P3-H5^T^. All data were from this study unless otherwise indicated. –, Not detected.

Cellular fatty acid (%)	1	2	3	4	5
Iso-C_14 : 0_	2.61	29.31	5.39	6.05	5.75
C_14 : 0_	0.39	–	0.31	0.32	0.5
Iso-C_15 : 0_	2.18	0.83	2.56	0.54	0.67
Anteiso-C_15 : 0_	64.20	43.31	53.38	60.26	78.85
C_16 : 1_* ω*7*c* alcohol	3.72	3.65	4.97	3.50	0.72
Iso-C_16 : 0_	1.05	13.77	9.62	5.31	2.61
C_16 : 1_* ω*11*c*	2.26	–	1.70	1.53	0.57
C_16 : 0_	1.40	0.64	1.25	1.34	1.39
Summed feature 4*	7.84	0.63	2.95	2.47	0.43
Summed feature 8*	0.33	–	–	–	–
C_17 : 1_* ω*9*c* anteiso	2.35	1.38	0.79	1.49	0.82
C_17 : 1_* ω*6*c*	2.40	0.31	0.46	0.41	
Iso-C_17 : 0_	0.22	–	0.68	–	–
Anteiso-C_17 : 0_	8.39	6.17	14.73	16.24	7.04
C_18 : 0_	0.65	–	0.19	–	0.3

*Summed features are fatty acids that cannot be resolved reliably from another fatty acid using the chromatographic conditions chosen. The midi system groups these fatty acids together as one feature with a single percentage of the total. Summed feature 4, iso-C_17 : 1_ I/anteiso-C_17 : 1_. Summed feature 8, C_18 : 1_* ω*7*c*/C_18 : 1_* ω*6*c.*

In summary, according to phylogenetic and phylogenomic data, strain C22-A2^T^ could be verified as representing a novel species of the genus *Virgibacillus.* In addition, the comparison of physiological and biochemical characteristics further distinguished it from related species of the genus *Virgibacillus*. Therefore, we propose that strain C22-A2^T^ represents a novel species of genus *Virgibacillus*, with the name *Virgibacillus tibetensis* sp. nov.

## Description of *Virgibacillus tibetensis* sp. nov.

*Virgibacillus tibetensis* (ti.bet.en’sis. N.L. masc. adj. *tibetensis*, of or belonging to Tibet, referring to the geographical origin of the type strain).

Cells are Gram-stain-negative, strictly aerobic, motile and long rod-shaped, 2.3 µm wide and 6.0–12 µm long. Colonies are circular, smooth, non-opaque, and white-coloured. Growth occurs at 4–30 °C (optimum, 20–25 °C), pH 7.0–10.0 (optimum, pH 8.0) and with 1.0–17.0% (w/v) NaCl (optimum, 3.0–7.0%). Catalase- and oxidase- positive. Hydrolyses starch, but not casein, cellulose, Tween 20 and Tween 80. DNase activity is not present. In the Biolog GEN III MicroPlate system, the type strain utilizes trehalose, *N*-acetyl-d-galactosamine, d-mannitol, d-arabitol, l-alanine, l-glutamic acid, l-serine, l-malic acid, acetoacetic acid, and acetic acid as sole carbon resources, and weakly utilizes dextrin, maltose, sucrose, *N*-acetyl-d-glucosamine, inosine, d-sorbitol, d-glucose-6-PO_4_, d-fructose-6-PO_4_, d-serine, l-arginine, l-aspartic acid, l-histidine, l-pyroglutamic acid, d-lactic acid metyl ester, α-keto-glutaric acid, d-malic acid, β-hydroxyl-d,l-butyric acid, and α-keto-butyric acid. In the API ZYM test, positive for alkaline phosphatase, esterase (C4), esterase lipase (C8), leucine arylamidase, α-chymotrypsin, naphthol-AS-BI-phosphohydrolase, and α-glucosidase. No acid is produced from any carbon source in API 50CH test. In API 20 NE and 20E tests, reduces nitrate to nitrite. Hydrolysis of gelatin is present. Voges–Proskauer test and activity of orthon nitrophenyl-*β*-d-galactopyranosidase and para-nitrophenyl-*β*-d-galactopyranosidase are positive. Citrate is not utilized. H_2_S and indole are not produced. Fermentation of glucose and hydrolysis of aesculin are not present. The activities of tryptophane deaminase, arginine dihydrolase, urease, lysine decarboxylase and ornithine decarboxylase are negative. The predominant cellular fatty acid is anteiso-C_15 : 0_ (64.2%). The major quinone is MK-7. The main polar lipids are phosphatidylglycerol, diphosphatidylglycerol and two unidentified lipids. The genomic DNA G+C content is 37.5%.

The type strain, C22-A2^T^ (=CGMCC 1.19202^T^=KCTC 43426^T^), was isolated from a water sample of salt lake LungmuCo in Tibet, PR China. The GenBank/EMBL/DDBJ accession numbers for the 16S rRNA gene and genome sequences of strain C22-A2^T^ are OR857398 and JARZFX000000000, respectively.

## supplementary material

10.1099/ijsem.0.006525 Uncited Supplementary Material 1.

## Abbreviations

ANI: average nucleotide identity
dDDH: digital DNA–DNA hybridization
LB: Luria–Bertani
MA: marine agar 2216
NA: nutrient agar
PYG: peptone–yeast–glucose
R2A: Reasoner’s 2A
TSA: tryptic soy agar

## References

[1] Heyndrickx M, Lebbe L, Kersters K, De Vos P, Forsyth G (1998). *Virgibacillus*: a new genus to accommodate *Bacillus pantothenticus* (Proom and Knight 1950). Emended description of *Virgibacillus pantothenticus*. Int J Sys Bacteriol.

[2] Niederberger TD, Steven B, Charvet S, Barbier B, Whyte LG (2009). *Virgibacillus arcticus* sp. nov., a moderately halophilic, endospore-forming bacterium from permafrost in the Canadian high Arctic. Int J Syst Evol Microbiol.

[3] Chen YG, Cui XL, Fritze D, Chai LH, Schumann P (2008). *Virgibacillus kekensis* sp. nov., a moderately halophilic bacterium isolated from a salt lake in China. Int J Syst Evol Microbiol.

[4] Wang CY, Chang CC, Ng CC, Chen TW, Shyu YT (2008). *Virgibacillus chiguensis* sp. nov., a novel halophilic bacterium isolated from Chigu, a previously commercial saltern located in southern Taiwan. Int J Syst Evol Microbiol.

[5] Kim J, Jung MJ, Roh SW, Nam YD, Shin KS (2011). *Virgibacillus alimentarius* sp. nov., isolated from a traditional Korean food. Int J Syst Evol Microbiol.

[6] Yoon JH, Kang SJ, Lee SY, Lee MH, Oh TK (2005). *Virgibacillus dokdonensis* sp. nov., isolated from a Korean island, Dokdo, located at the edge of the East Sea in Korea. Int J Syst Evol Microbiol.

[7] Denariaz G, Payne WJ, Le Gall J (1989). A halophilic denitrifier, *Bacillus halodenitrificans* sp. nov. Int J Syst Bacteriol.

[8] Yoon JH, Oh TK, Park YH (2004). Transfer of *Bacillus halodenitrificans* Denariaz *et al*. 1989 to the genus *Virgibacillus* as *Virgibacillus halodenitrificans* comb. nov. Int J Syst Evol Microbiol.

[9] Karlson U, Dwyer DF, Hooper SW, Moore ER, Timmis KN (1993). Two independently regulated cytochromes P-450 in a *Rhodococcus rhodochrous* strain that degrades 2-ethoxyphenol and 4-methoxybenzoate. J Bacteriol.

[10] Lane DJ, Goodfellow M, Stackebrandt E (1991). Nucleic Acid Techniques in Bacterial Systematics.

[11] Yoon S-H, Ha S-M, Kwon S, Lim J, Kim Y (2017). Introducing EzBioCloud: a taxonomically united database of 16S rRNA gene sequences and whole-genome assemblies. Int J Syst Evol Microbiol.

[12] Kumar S, Stecher G, Li M, Knyaz C, Tamura K (2018). MEGA X: Molecular Evolutionary Genetics Analysis across computing platforms. Mol Biol Evol.

[13] Larkin MA, Blackshields G, Brown NP, Chenna R, McGettigan PA (2007). Clustal W and Clustal X version 2.0. Bioinformatics.

[14] Kimura M (1980). A simple method for estimating evolutionary rates of base substitutions through comparative studies of nucleotide sequences. J Mol Evol.

[15] Saitou N, Nei M (1987). The neighbor-joining method: a new method for reconstructing phylogenetic trees. Mol Biol Evol.

[16] Felsenstein J (1981). Evolutionary trees from DNA sequences: a maximum likelihood approach. J Mol Evol.

[17] Fitch WM (1971). Toward defining the course of evolution: minimum change for a specific tree topology. Syst Zool.

[18] Wick RR, Judd LM, Gorrie CL, Holt KE (2017). Unicycler: resolving bacterial genome assemblies from short and long sequencing reads. PLoS Comput Biol.

[19] Davis JJ, Wattam AR, Aziz RK, Brettin T, Butler R (2020). The PATRIC bioinformatics resource center: expanding data and analysis capabilities. Nucleic Acids Res.

[20] Yoon SH, Ha SM, Lim JM, Kwon SJ, Chun J (2017). A large-scale evaluation of algorithms to calculate average nucleotide identity. Antonie van Leeuwenhoek.

[21] Meier-Kolthoff JP, Auch AF, Klenk HP, Göker M (2013). Genome sequence-based species delimitation with confidence intervals and improved distance functions. BMC Bioinformatics.

[22] Rodriguez-R LM, Konstantinidis KT (2014). Bypassing cultivation to identify bacterial species. Microbe Magazine.

[23] Carrasco IJ, Márquez MC, Ventosa A (2009). *Virgibacillus salinus* sp. nov., a moderately halophilic bacterium from sediment of a saline lake. Int J Syst Evol Microbiol.

[24] Xu B, Hu B, Wang J, Lan Y, Zhu Y (2018). *Virgibacillus indicus* sp. nov. and *Virgibacillus profundi* sp. nov, two moderately halophilic bacteria isolated from marine sediment by using microfluidic streak plates. Int J Syst Evol Microbiol.

[25] Amziane M, Metiaz F, Darenfed-Bouanane A, Djenane Z, Selama O (2013). *Virgibacillus natechei* sp. nov., a moderately halophilic bacterium isolated from sediment of a saline lake in southwest of Algeria. Curr Microbiol.

[26] Aziz RK, Bartels D, Best AA, DeJongh M, Disz T (2008). The RAST server: rapid annotations using subsystems technology. BMC Genom.

[27] Overbeek R, Olson R, Pusch GD, Olsen GJ, Davis JJ (2014). The SEED and the Rapid Annotation of microbial genomes using Subsystems Technology (RAST). Nucl Acids Res.

[28] Blin K, Shaw S, Augustijn HE, Reitz ZL, Biermann F (2023). antiSMASH 7.0: new and improved predictions for detection, regulation, chemical structures and visualisation. Nucleic Acids Res.

[29] McArthur AG, Waglechner N, Nizam F, Yan A, Azad MA (2013). The comprehensive antibiotic resistance database. Antimicrob Agents Chemother.

[30] Hegemann JD, Zimmermann M, Xie X, Marahiel MA (2015). Lasso peptides: an intriguing class of bacterial natural products. Acc Chem Res.

[31] Maksimov MO, Pan SJ, James Link A (2012). Lasso peptides: structure, function, biosynthesis, and engineering. Nat Prod Rep.

[32] Zhu S, Hegemann JD, Fage CD, Zimmermann M, Xie X (2016). Insights into the unique phosphorylation of the lasso peptide paeninodin. J Biol Chem.

[33] Kuhlmann AU, Bremer E (2002). Osmotically regulated synthesis of the compatible solute ectoine in *Bacillus pasteurii* and related *Bacillus* spp. Appl Environ Microbiol.

[34] Gerhardt P, Murray RGE, Wood WA, Krieg NR (1994). Methods for General and Molecular Bacteriology.

[35] Miller LT (1982). Single derivatization method for routine analysis of bacterial whole-cell fatty acid methyl esters, including hydroxy acids. J Clin Microbiol.

[36] Sasser M (1990). MIDI Technical Note 101.

[37] Minnikin DE, Bolton RC, Hartmann S, Besra GS, Jenkins PA (1993). An integrated procedure for the direct detection of characteristic lipids in tuberculosis patients. Ann Soc Belg Med Trop.

[38] Collins MD, Goodfellow M, Minnikin DE (1985). Chemical Methods in Bacterial Systematics.

[39] Wu C, Lu X, Qin M (1989). Analysis of menaquinone compound in microbial cells by HPLC. Microbiology.

