# Supplementary material for: Virgibacillus tibetensis sp. nov., isolated from salt lake on the Tibetan plateau of China
Source: Int J Syst Evol Microbiol. 2024 Sep 23;74(9):006525. doi: 10.1099/ijsem.0.006525 (PMC11419321; doi:10.1099/ijsem.0.006525)
Supplement: Uncited Supplementary Material 1. [file ijsem-74-06525-s001.pdf]

Virgibacillus tibetensis sp. nov., isolated from salt lake on the Tibetan Plateau of China

Dorji Phurbu[1], Xuan Zhang[2,4], Zi-Xuan Liu[2], Rui Wang[3], Yan-Yan Zheng[1], Hong-Can Liu[2], Yu-Guang Zhou[2], Ya-Jing Yu[2], Ai-Hua Li[2*]

1. Tibet Plateau Key Laboratory of Mycology, Tibet Plateau Institute of Biology, Lhasa, Tibet 850001, PR China;
2. China General Microbiological Culture Collection Center (CGMCC), Institute of Microbiology, Chinese Academy of Sciences, Beijing 100101, PR China;
3. Tianjin Institute of Industrial Biotechnology, Chinese Academy of Sciences, Tianjin 300308, PR China;
4. School of Biotechnology and Food Science, Tianjin University of Commerce, Tianjin, PR China

Corresponding author:

*Ai-Hua Li

Email: liah@im.ac.cn; Tel: 86-10-6480-6073; Fax: 86-10-6480-7288

Running title: *Virgibacillus tibetensis* from salt lake

Subject category: New Taxa; **Subsection:** *Bacillales*

Keywords: *Virgibacillus*, Tibetan Plateau, salt lake, *Bacillaceae*

Table S1：General Characteristics of Genome

Strain	C22-A2^{T}
16S rRNA	OR857398
Accession number	JARZFX000000000
**Sequence information**	
Genome size（bp）	4291980
Number of Scaffolds	43
G+C（%）	37.5 %
N_{50}（bp）	318248
**Annotation information**	
CDSs	4197
rRNA	4
tRNA	61
**Protein features**	
Hypothetical proteins	83
Proteins with functional assignments	2779
Proteins with EC number assignments	923
Proteins with GO assignments	791
Proteins with Pathway assignments	707
Proteins with Subsystem assignments	1100
Proteins with PATRIC genus-specific family (PLfam) assignments	2345
Proteins with PATRIC cross-genus family (PGfam) assignments	2903
Proteins with FIGfam assignments	0
**Specialty Genes**	
Transporter TCDB	3
Drug Target DrugBank	3
Antibiotic Resistance PATRIC	34
Antibiotic Resistance CARD	1

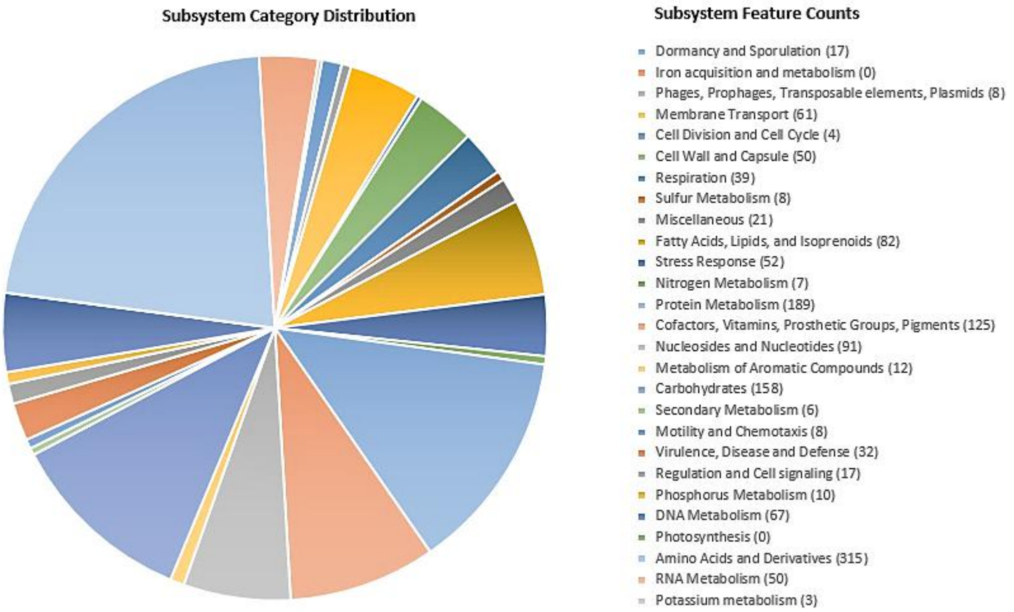

Figure S1. The subsystem category number of genes in strain C22-A2^T detected by RAST annotation server.

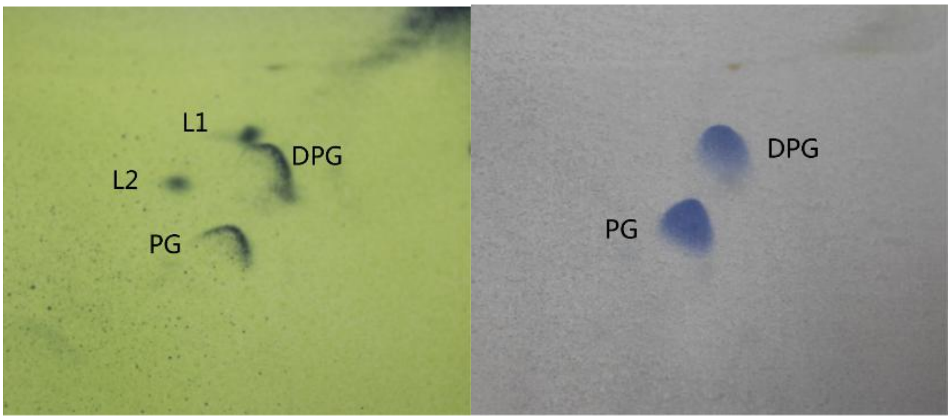

a b

Figure S2. a: Two-dimensional TLC of the total polar lipids of strain C22-A2^T with ethanolic phosphomolybdic acid. b: Phospholipids of strain C22-A2^T with anisaldehyde. (PG, phosphatidylglycerol; DPG, diphosphatidylglycerol; L1-2, unidentified lipid).
